# An epigenome-wide association study of metabolic syndrome and its components

**DOI:** 10.1038/s41598-020-77506-z

**Published:** 2020-11-25

**Authors:** Marja-Liisa Nuotio, Natalia Pervjakova, Anni Joensuu, Ville Karhunen, Tero Hiekkalinna, Lili Milani, Johannes Kettunen, Marjo-Riitta Järvelin, Pekka Jousilahti, Andres Metspalu, Veikko Salomaa, Kati Kristiansson, Markus Perola

**Affiliations:** 1 Institute for Molecular Medicine Finland (FIMM), University of Helsinki, Helsinki, Finland; 2 Genomics and Biobank Unit, Department of Public Health Solutions, National Institute for Health and Welfare, Biomedicum 1, Haartmaninkatu 8, 00290 Helsinki, Finland; 3 Research Program for Clinical and Molecular Metabolism, Faculty of Medicine, University of Helsinki, Helsinki, Finland; 4 Estonian Genome Center, Institute of Genomics, University of Tartu, Tartu, Estonia; 5 Department of Biotechnology, Institute of Molecular and Cell Biology, University of Tartu, Tartu, Estonia; 6 Center for Life Course Health Research, University of Oulu, Oulu, Finland; 7 Oulu University Hospital, Oulu, Finland; 8 Biocenter Oulu, University of Oulu, Oulu, Finland; 9 Computational Medicine, Faculty of Medicine, University of Oulu, Oulu, Finland; 10 Population Health Science, Bristol Medical School, University of Bristol and Medical Research Council Integrative Epidemiology Unit, University of Bristol, Bristol, UK; 11 Unit of Primary Health Care, Oulu University Hospital, Oulu, Finland; 12 Department of Epidemiology and Biostatistics, MRC-PHE Centre for Environment and Health, School of Public Health, Imperial College London, London, UK; 13 Finnish Institute for Health and Welfare, Helsinki, Finland

**Keywords:** DNA methylation, Genetics research

## Abstract

The role of metabolic syndrome (MetS) as a preceding metabolic state for type 2 diabetes and cardiovascular disease is widely recognised. To accumulate knowledge of the pathological mechanisms behind the condition at the methylation level, we conducted an epigenome-wide association study (EWAS) of MetS and its components, testing 1187 individuals of European ancestry for approximately 470 000 methylation sites throughout the genome. Methylation site cg19693031 in gene *TXNIP* —previously associated with type 2 diabetes, glucose and lipid metabolism, associated with fasting glucose level (*P* = 1.80 × 10^−8^). Cg06500161 in gene *ABCG1* associated both with serum triglycerides (*P* = 5.36 × 10^−9^) and waist circumference (*P* = 5.21 × 10^−9^). The previously identified type 2 diabetes–associated locus cg08309687 in chromosome 21 associated with waist circumference for the first time (*P* = 2.24 × 10^−7^). Furthermore, a novel HDL association with cg17901584 in chromosome 1 was identified (*P* = 7.81 × 10^−8^). Our study supports previous genetic studies of MetS, finding that lipid metabolism plays a key role in pathology of the syndrome. We provide evidence regarding a close interplay with glucose metabolism. Finally, we suggest that in attempts to identify methylation loci linking separate MetS components, cg19693031 appears to represent a strong candidate.

## Introduction

Metabolic syndrome (MetS) is a biologically complex condition encompassing a cluster of risk factors such as central obesity, elevated triglycerides, reduced HDL cholesterol, elevated fasting plasma glucose, and elevated blood pressure^1^. When co-occurring, these risk factors create a 1.7- to 1.9-fold risk for cardiovascular disease and a 3.0- to 6.1-fold risk for type 2 diabetes mellitus compared to individuals without the syndrome^2^. The globally increasing prevalence of type 2 diabetes and cardiovascular disease associated with the underlying obesity pandemic emphasises the importance of efforts aimed at unravelling the complicated biological mechanisms of MetS.

The underlying pathogenesis for MetS as a condition is not completely understood. To date, genetic study methods including candidate gene studies, linkage studies, and genome-wide association studies (GWAS) have enjoyed modest success in finding genetic associations with MetS when treated as a dichotomous trait. By contrast, the majority of the associations identified relate to one of the individual components of MetS with variable pleiotropy accompanying other components^3–7^. These findings have made it reasonable to hypothesize that MetS may not be a single entity at the genomic level and further raised a question if a specific genomic area that would be jointly associated to all of these component phenotypes could be found^6^. In previous GWA- studies the role of lipid metabolism as a major contributor to the MetS phenotypic outcome has emphasised^6^.

Epigenetics might serve as one of the factors linking the individual components of the MetS phenotypic outcome. Thus far, only a few studies have successfully identified epigenetic regions and individual markers associated with separate MetS components and only four epigenetic markers have been reported to be associated with MetS as a whole^8–10^.

We therefore conducted an epigenome-wide association study (EWAS) of MetS and its components using whole blood methylation levels in order to test the association (i) between methylation (CpG) and MetS as a binomial trait, as well as (ii) between methylation (CpG) and individual MetS components to shed light on the metabolic interplay underlying this complex phenotype.

## Materials and methods

### Study population

Our study included two populations of Finnish ancestry: a discovery cohort consisting of 517 individuals (the National FINRISK Study, DILGOM), as well as a replication cohort consisting of 670 individuals (the Northern Finland Birth Cohort 1966). Local research ethics committees approved both studies (Coordinating Ethics Committee of the HUS Hospital District for DILGOM, decision numbers 229/E0/2006 and 332/13/03/00/2013, respectively and The Ethical Committee of the Northern Ostrobothnia Hospital District for NFBC1966, decision number 1/2012). All experiments were performed in accordance with relevant guidelines and regulations.

### The national FINRISK study

FINRISK surveys consist of cross-sectional, population-based studies conducted every five years between 1972 and 2012 to monitor the risk of chronic diseases in Finland. Each survey included a questionnaire and a clinical examination during which a blood sample was drawn. Data are linked to the national electronic healthcare registers for cardiovascular disease and other health outcomes^11^.

Our study consisted of eligible individuals from a specific subset examined for metabolic traits during the 2007 FINRISK survey. The DILGOM study (The Dietary, Lifestyle, and Genetic Determinants of Obesity and Metabolic Syndrome) was collected as an extension of the FINRISK 2007 survey. This subset consisted of a total sample size of 5025 individuals aged between 25 and 74, of whom we studied 517 individuals from the Helsinki and Vantaa region using various omics^12^. Detailed characteristics of the study sample are provided in Table 1. Detailed descriptions of the methodologies used to measure the waist circumference (WC), triglycerides, HDL, systolic and diastolic blood pressure, and plasma glucose levels for the study sample are described in Supplementary material as well as in previous studies^13,14^. In this study, the presence of MetS is defined according to the 2005 International Diabetes Federation (IDF) definition (Table 2)^1^.

**Table 1:** Sample characteristics.

	FINRISK 2007 (DILGOM)			NFBC1966		
	All*	MetS cases	MetS controls	All	MetS cases	MetS controls
N	517	212	284	670	57	613
Age (years)	51.9 (13.8)	57.9 (11.5)	47.1 (13.4)	31.0 (0.3)	31.0 (0.3)	31.0 (0.3)
Sex (% males)	46.2	50.9	43.0	45.1	61.4	43.6
MetS case (%), (males / females)	42.5 (47.0 / 38.7)	-	-	8.5 (11.6 / 6.0)	-	-
Current smokers (%)	19.5	19.8	19.7	21.9	28.1	21.4
Alcohol use (g/week)	78.2 (109.6)	81.6 (111.2)	78.5 (110.7)	61.5 (101.0)	89.4 (105.9)	58.9 (100.2)
Waist circumference (cm), (males / females)	97.1 (11.2) / 87.1 (13.7)	105.1 (8.0)/ 97.5 (12.9)	90.1 (8.6)/ 80.6 (9.6)	88.5 (9.7) / 77.9 (10.5)	102.1 (7.1) / 98.6 (12.5)	86.7 (8.5) /76.6 (8.8)
Triglycerides (mmol/l)	1.3 (0.6)	1.6 (0.7)	1.1 (0.5)	1.1 (0.7)	2.1 (1.0)	1.0 (0.6)
HDL (mmol/l)	1.5 (0.4)	1.3 (0.3)	1.6 (0.4)	1.6 (0.4)	1.3 (0.3)	1.6 (0.4)
Systolic blood pressure (mmHg)	132.0 (17.9)	139.8 (16.1)	126.0 (16.8)	124.7 (13.7)	137.3 (14.9)	123.5 (13.0)
Diastolic blood pressure (mmHg)	79.4 (10.3)	82.8 (9.9)	76.9 (9.8)	77.1 (11.4)	88.6 (12.5)	76.0 (10.7)
Fasting plasma glucose (mmol/l)	5.8 (0.7)	6.3 (0.7)	5.5 (0.5)	5.0 (0.8)	5.5 (0.7)	5.0 (0.8)

Data is given as mean (SD) or %.

*DILGOM* dietary, lifestyle, and genetic determinants of obesity and metabolic syndrome, *NFBC1966* Northern Finland Birth Cohort 1966, *MetS* metabolic syndrome, *HDL* high-density lipoprotein.

*The number of study participants in DILGOM refers to the individuals for whom methylation data were available. Sample sizes vary between analysed traits as described in Supplementary material.

**Table 2:** Definition of metabolic syndrome (MetS) according to the International Diabetes Federation (IDF).

Central obesity measured as waist circumference (WC) (≥ 94 cm for men / ≥ 80 cm for women)	
Plus any two:	
Triglycerides	≥ 1.7 mmol/l or medication for elevated triglycerides
HDL	< 1.03 mmol/l in men and < 1.3 mmol/l in women or a medication to reduce HDL cholesterol
Blood pressure	≥ 130 mmHg (systolic) or ≥ 85 mmHg (diastolic) or a diagnosis or medication for hypertension
Fasting plasma glucose	≥ 5.6 mmol/l or a diagnosis or medication for type 2 diabetes

*HDL* high-density lipoprotein.

### The Northern Finland Birth Cohort 1966 (NFBC1966)

The Northern Finland Birth Cohort 1966 (NFBC1966) is a population-based prospective birth cohort consisting of all mothers in the two northern-most provinces of Finland with children whose expected date of birth fell during 1966^15^. In total, 12 058 live-born children were recruited into the cohort. In 1997, a health and lifestyle questionnaire was sent to all living cohort members with a known address (*n* = 11 322). Those cohort members living in Northern Finland and in the Helsinki area (*n* = 8463) were also invited for a clinical examination, of whom 6033 attended. Information on smoking and alcohol use was obtained from the questionnaire. Descriptions of the methodologies used to measure the waist circumference (WC), triglycerides, HDL, systolic and diastolic blood pressure, and plasma glucose levels for the study sample are described in detail in Supplementary material. Informed consent and written permission were obtained from the study participants at both 31 and 46 years of age.

### DNA extraction and methylation array analysis

In the DILGOM cohort, whole blood samples were obtained from 517 individuals (*n* = 239 men and *n* = 278 women). All subjects were older than 18 years, with a mean age of 51.9 years, and provided their written informed consent for the use of their DNA sample for research.

DNA was extracted from 10-ml whole-blood peripheral white blood cells using the NucleoSpin® Tissue kit (Macherey–Nagel GmbH, Düren, Germany) with the salting-out method using 10 M ammonium acetate. DNA was precipitated in isopropanol, washed in 70% ethanol, and resuspended in 1X TE buffer. The purity and concentrations of the DNA samples were measured by spectrophotometer (NanoDrop® ND1000; Thermo Fisher Scientific Inc., Waltham, MA, US). From each sample, 600 ng of genomic DNA was bisulfite modified using the EZ DNA Methylation kit (Zymo Research Orange, California, US) according to the manufacturer’s recommendations for the Illumina Infinium Assay. After purification, 4 µl of each bisulfite-converted DNA sample was used for hybridisation on the Infinium Human Methylation 450 BeadChip (HM450K) following the Illumina Infinium HD Methylation protocol with the original IDAT files extracted from the HiScan scanner.

In NFBC1966, DNA methylation was extracted after a 31-year follow-up period for a random sample of 807 participants for whom complete follow-up data were available (postal questionnaire and clinical examination at 31 as well as 46 years of age). DNA methylation was further measured using the HM450K assay.

### Methylation normalisation

In the DILGOM cohort, the methylation data processing and quality control analyses were performed using the Bioconductor package *minfi*^16^. Pre-normalised raw data were used to convert the intensities from the red and the green channels into methylated (M) and unmethylated (U) signals. Βeta values for each CpG probe were calculated according to Illumina’s recommendations using [β = M/(M + U + 100)]. The difference in the distribution of β values for type I and type II probes was corrected using subset-quantile within array normalisation (SWAN)^17^. Detection *P* values were obtained for every CpG probe in every sample. Failed positions were defined as signal levels lower than background from both methylated and unmethylated channels. Probes with a detectable methylation level in < 5% of samples (detection *P* < 0.01) were excluded. In addition, the CpG sites on the X and Y chromosomes were discarded, which resulted in 468 809 CpG probes for further analysis.

In NFBC1966, quality control and normalisation for DNA methylation data were performed based on the CPACOR pipeline^18^ with minor adaptations. The pipeline uses 30 PCs as covariates to control for technical confounding. Data were first retrieved using the *minfi* R package^16^, and the Illumina Background Correction to the intensity values was applied. A detection *P* value threshold was set to *P* < 1 × 10^−16^, and samples with a call rate < 98% were excluded. Quantile normalisation was performed separately for six probe-type categories using R package *limma*^19^. These normalised intensity values were used to calculate the β value at each CpG site. A principal component analysis was performed for HM450K control probes, and the first 30 principal components were used as additional explanatory variables in subsequent regression models in both, DILGOM and NFBC1966 cohorts. In both DILGOM and NFBC1966, white blood cell subpopulation estimates were acquired using the software provided by Houseman et al.^20^, and these subpopulation estimates were also added to regression models as explanatory variables. Further exclusions of individuals from DILGOM and NFBC1966 data were performed before association analyses, based on outlier-status and gender mismatches (Supplementary material).

### Power calculation

Power calculation for DILGOM cohort was performed with pwrEWAS^21^. For binary analyses within the discovery sample of approximately 500 individuals, differences up to 20% in CpG-specific methylation with at 94% power and differences up to 2% at 28% power were able to be detected.

### Epigenome-wide association study (EWAS)

In the discovery analysis of the DILGOM cohort, the association between the M value for all 468 809 CpG probes (as outcome variable) and MetS, as well as its six individual components, were tested using regression analysis fitting generalised linear models (*glm*) using the R software program, version 3.3.1^22^. Analyses were adjusted for age, sex, smoking status (defined either as current or never/ex-smokers), alcohol consumption (grams/week), cell subtype proportion, study- specific technical covariates (described in detail in Supplementary material), and the first five genetic principal components of the data to control for potential population substructure.

### Replication analysis

Association analysis of a total of 33 CpG probes was replicated in an independent population sample (NFBC1966, *n* = 670, Supplementary Table 1, Supplementary Fig. 1). The 33 CpG probes were selected for replication by generating Q–Q probability plots of the discovery EWAS results of MetS and its individual components and further verifying selected probes not to be false positive discoveries using permutation analysis. The EWAS results of those 33 CpG probes clearly deviated from the expected line of the Q–Q probability plots. In permutation analysis of each probe we first 1) randomly re-assigned the probe signal values between the study participants while keeping their phenotype data untouched, and then 2) tested for an association between the probe and the phenotypes as described above for the original association analysis. We repeated this procedure 1000 times and calculated how many occurrences of a *P* value smaller than the *P* value from the original association analysis was observed. For all 33 selected probes, the count was less than 1%. The replication analysis for the 33 CpG probes was performed using the same analytical protocol used in the discovery analysis for the DILGOM cohort.

### Meta-analysis

Summary statistics from both cohorts were meta-analysed using an inverse-variance weighted fixed-effect model, using the GWAMA software program^23^.

### Conditional analysis

To investigate the independence of the replicated methylation signals from the possible underlying genetic effect (DNA variation expressed as an SNP effect), a conditional linear regression analysis was performed for selected, successfully replicated probes (N = 5). First, SNPs located ± 5 Mb from the genomic position of each replicated methylation probe were tested for an association with the phenotype in question (Supplementary Fig. 2). The strongest associating SNP (based on their association *P *value) was then used as a covariate in the regression analysis where association between the methylation probe and phenotype was tested.

### Gene expression data processing and analysis

Sample collection and data processing is described in detail in Inouye et al.^24^. In brief, RNA was hybridized to Illumina HT-12 v3 BeadChip arrays. The background corrected probes were subjected to quantile normalization for each array at the strip-level. Technical replicates were combined by bead count weighted average and replicates with Pearson correlation coefficient < 0.94 or Spearman’s rank correlation coefficient < 0.60 were removed. Expression values for each probe were log2 transformed.

To investigate the relation between the replicated methylation signals and gene expression, a linear regression analysis was performed for successfully replicated methylation probes located to a gene and equivalent gene transcripts (available for three replicated methylation loci). The same covariates were used to adjust the analyses as those used in discovery EWA analyses. Possible *trans* effects between selected replicated methylation loci and nearest gene transcripts of other replicated methylation probes were also studied with linear regression analysis.

## Results

### Epigenome-wide association between DNA methylation and MetS and its components

**Figure 1:**
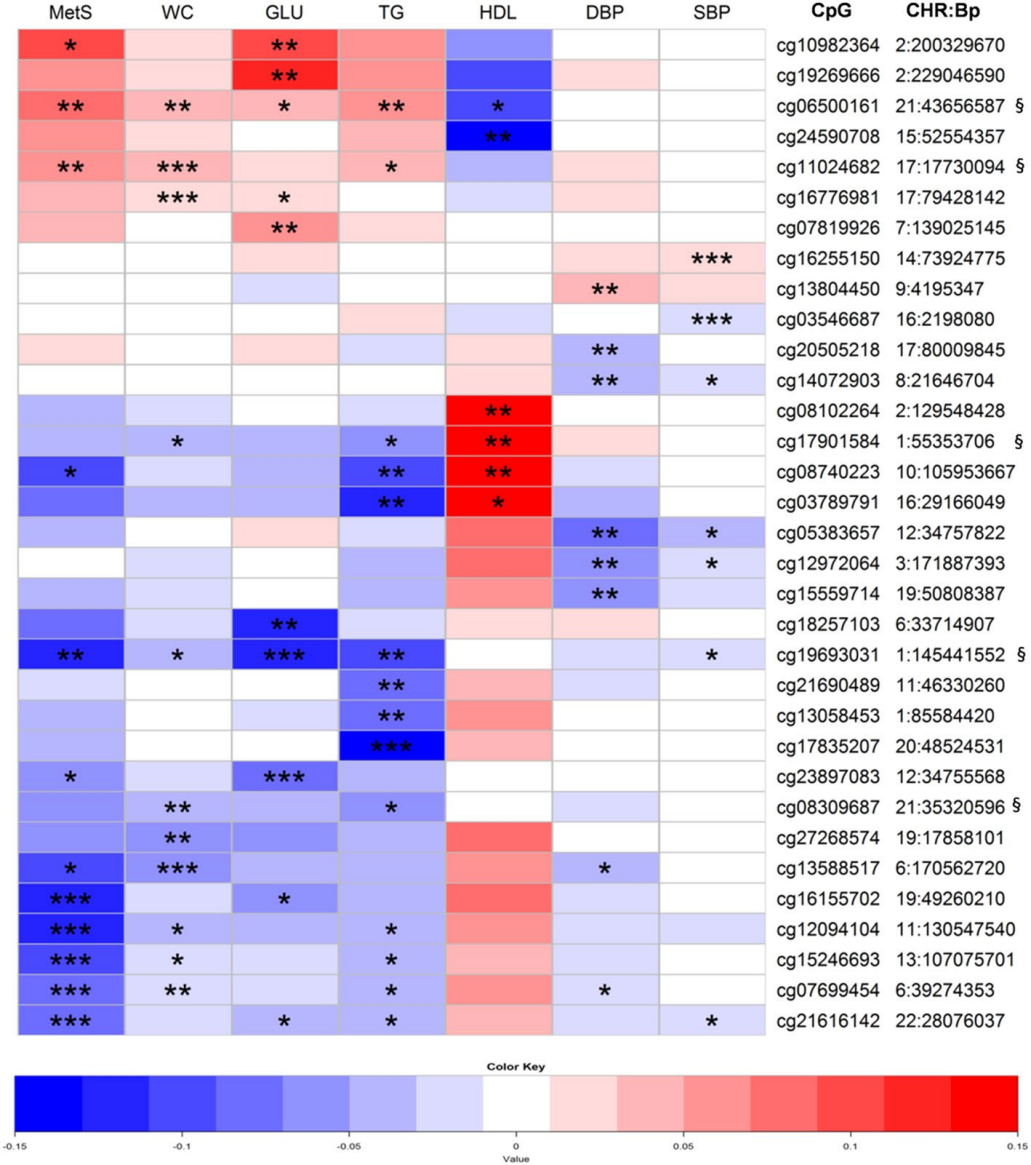
Association of CpG methylation sites across metabolic syndrome (MetS) and related phenotypes in the DILGOM cohort. The heat map includes the 33 most significant probes from the discovery epigenome-wide association studies (EWAS). Colours in the map refer to the effect size (beta) values for the probe in question. Beta values exceeding 0.15 are recoded as 0.15 and values below –0.15 are recoded as –0.15 in the figure. Stars indicate the *P* values from individual tests as follows: *P < 0.01, **P < 0.0001, ***P < 1 × 10–6. Beta values from the waist circumference, diastolic blood pressure, and systolic blood pressure analyses are multiplied by 10 in order to provide more comparable and clinically relevant values for beta in the heat map. The detailed numeric data for the heat map is presented in the Supplementary Table 1. § indicates the successful replication of the association between the methylation probe and the individual phenotype. *MetS* metabolic syndrome as case/control, *WC* waist circumference, *GLU* fasting glucose, *HDL* high-density lipoprotein, *TG* triglycerides, *DBP* diastolic blood pressure, *SBP* systolic blood pressure, *CpG* cytosine–guanine dinucleotide, *CHR* chromosome, *Bp* base pair.

We found several differentially methylated CpG methylation sites for MetS and its component traits (WC, fasting glucose, HDL, triglycerides, systolic blood pressure, diastolic blood pressure) across the 468 809 CpG probes analysed in the FINRISK sample on 517 Finnish individuals. In total, 33 probes with methylation M value based *P* values ranging from *P* = 7.33 × 10^−6^ to *P* = 5.08 × 10^−8^, passed permutation based test of statistical significance (*P*_perm_ < 0.05) and clearly deviated from the expected line of the Q–Q probability plots of the discovery analysis results (Supplementary Fig. 1). From these, 12 probes associated directly with the MetS status, and all 33 associated with one or more component traits. The 33 probes were further selected for a replication analysis (Fig. 1, Supplementary Table 1).

### Replication, meta-analysis and integration with genetics

Altogether, five CpG markers were successfully replicated in an independent Finnish sample of 670 individuals at a nominal significance level of *P* < 0.05 (Table 3), and subsequently tested in a meta-analysis combining the discovery and replication cohorts. All replicated associations were for the component phenotypes and none for the MetS case/control status. The methylation site cg19693031 in the *TXNIP* gene region (chr1:145 441 552) associated inversely with fasting glucose levels in the meta-analysis (β_eff_ = –0.076, *P* = 1.80 × 10^−8^). The methylation site cg06500161 in the ATP-binding cassette, sub-family G (WHITE), member 1 (*ABCG1*) gene region (chr21:43 656 587) associated both with serum triglycerides (β_eff_ = 0.047, *P* = 5.36 × 10^−9^) and WC (β_eff_ = 0.003, *P* = 5.21 × 10^−9^). Two other WC–associated methylation sites were also observed: cg08309687, located within the long intergenic non-protein coding RNA 649 approximately 8 Mb downstream from the *ABCG1* gene region (β_eff_ = –0.004, *P* = 2.24 × 10^−7^), and cg11024682 in the sterol regulatory element binding transcription factor 1 *(SREBF1*) gene region (chr17:17 730 094) (β_eff_ = 0.003, *P* = 5.96 × 10^−9^). Furthermore, methylation site cg17901584 located in the genomic region of *HP08874* mRNA (chr1:55 353 706) associated with HDL (β_eff_ = 0.133, *P* = 7.81 × 10^−8^).

**Table 3:** Successfully replicated CpG M value—phenotype associations and the meta-analysis.

Phenotype	CpG	Position (Chr:bp) GRCh37	Gene	DILGOM				NFBC1966				Meta-analysis (DILGOM and NFBC1966)			
				*n*	Effect	SE	*P*	*n*	Effect	SE	*P*	*n*	Effect	SE	*P*
Fasting glucose	cg19693031	1:145,441,552	*TXNIP*	498	− 0.123	0.023	8.47E−08	670	− 0.050	0.017	0.003	1168	−0.076	0.013	1.80E−08
HDL	cg17901584	1:55,353,706	*DHCR24 / HP08874* mRNA, partial sequence	498	0.156	0.034	4.63E−06	670	0.106	0.037	0.004	1168	0.133	0.025	7.81E−08
Triglycerides	cg06500161	21:43,656,587	*ABCG1*	498	0.069	0.015	3.49E−06	670	0.038	0.010	1.10E−04	1168	0.047	0.008	5.36E−09
WC	cg11024682	17:17,730,094	*SREBF1*	497	0.003	0.001	1.15E−07	670	0.002	0.001	0.006	1167	0.003	0.0004	5.96E−09
	cg06500161	21:43,656,587	*ABCG1*	497	0.004	0.001	1.75E−06	670	0.002	0.001	4.74E−04	1167	0.003	0.0005	5.21E−09
	cg08309687	21:35,320,596	long intergenic non-protein coding RNA 649	497	− 0.005	0.001	1.85E−06	670	− 0.003	0.001	0.023	1167	−0.004	0.001	2.24E−07

*DILGOM* dietary, lifestyle, and genetic determinants of obesity and metabolic syndrome, *NFBC1966* Northern Finland Birth Cohort 1966, *HDL* high-density lipoprotein, *WC* waist circumference, *CpG* cytosine–guanine dinucleotide, *Chr* chromosome, bp, base pair, *GRCh37* the Genome Reference Consortium human genome (build 37), *SE* standard error.

### Similarity among the association results of component phenotypes of MetS

Notable similarity among the association results of the component phenotypes building up the metabolic syndrome as an entity was observed for the 33 CpG probes taken forward for replication (Fig. 1). Several methylation probes associated with both WC and MetS (Fig. 1, Suppl. Table 1). Numerous methylation probes associated with triglycerides and WC, often accompanied by a concurrent association with MetS. Unsurprisingly, several probes were also simultaneously associated both with triglycerides and HDL as well as both with glucose and MetS (Fig. 1).

Two of the five replicated methylation probes simultaneously associated with MetS and with several of its components in the discovery cohort. In addition to the association with triglycerides and WC, methylation site cg06500161 in the *ABCG1* gene associated with glucose, HDL, and MetS (β_eff_ = 0.049, *P* = 7.39 × 10^−4^; β_eff_ = –0.096, *P* = 2.70 × 10^−4^; β_eff_ = 0.077, *P* = 7.44 × 10^−5^, respectively). Furthermore, the glucose-associated methylation site cg19693031 in the *TXNIP* gene region suggestively associated with WC, triglycerides, systolic blood pressure as well as MetS as a condition (β_eff_ = –0.004, *P* = 2.25 × 10^−3^; β_eff_ = –0.107, *P* = 1.09 × 10^−5^; β_eff_ = –0.003, *P* = 3.24 × 10^−3^; β_eff_ = –0.124, *P* = 6.98 × 10^−5^, respectively), but notably not with HDL (Fig. 1, Suppl. Table 1).

In addition, the remaining replicated methylation probes associated with a few other MetS component phenotypes as follows. The WC-associated site cg08309687, located downstream from the *ABCG1* gene region, also associated with triglycerides (β_eff_ = –0.058, *P* = 4.17 × 10^−3^). Cg11024682 in the *SREBF1* gene region appeared to associate with triglycerides and MetS (β_eff_ = 0.034, *P* = 5.21 × 10^−3^; β_eff_ = 0.067, *P* = 2.11 × 10^−5^, respectively). Despite the general association pattern with the lipid components of MetS, neither cg08309687 nor cg11024682 associated with HDL. Rather, HDL-associated site cg17901584 appeared to also associate with triglycerides and WC (β_eff_ = –0.056, *P* = 4.44 × 10^−3^; β_eff_ = –0.003, *P* = 1.28 × 10^−3^, respectively) (Fig. 1, Suppl. Table 1).

**Figure 2:**
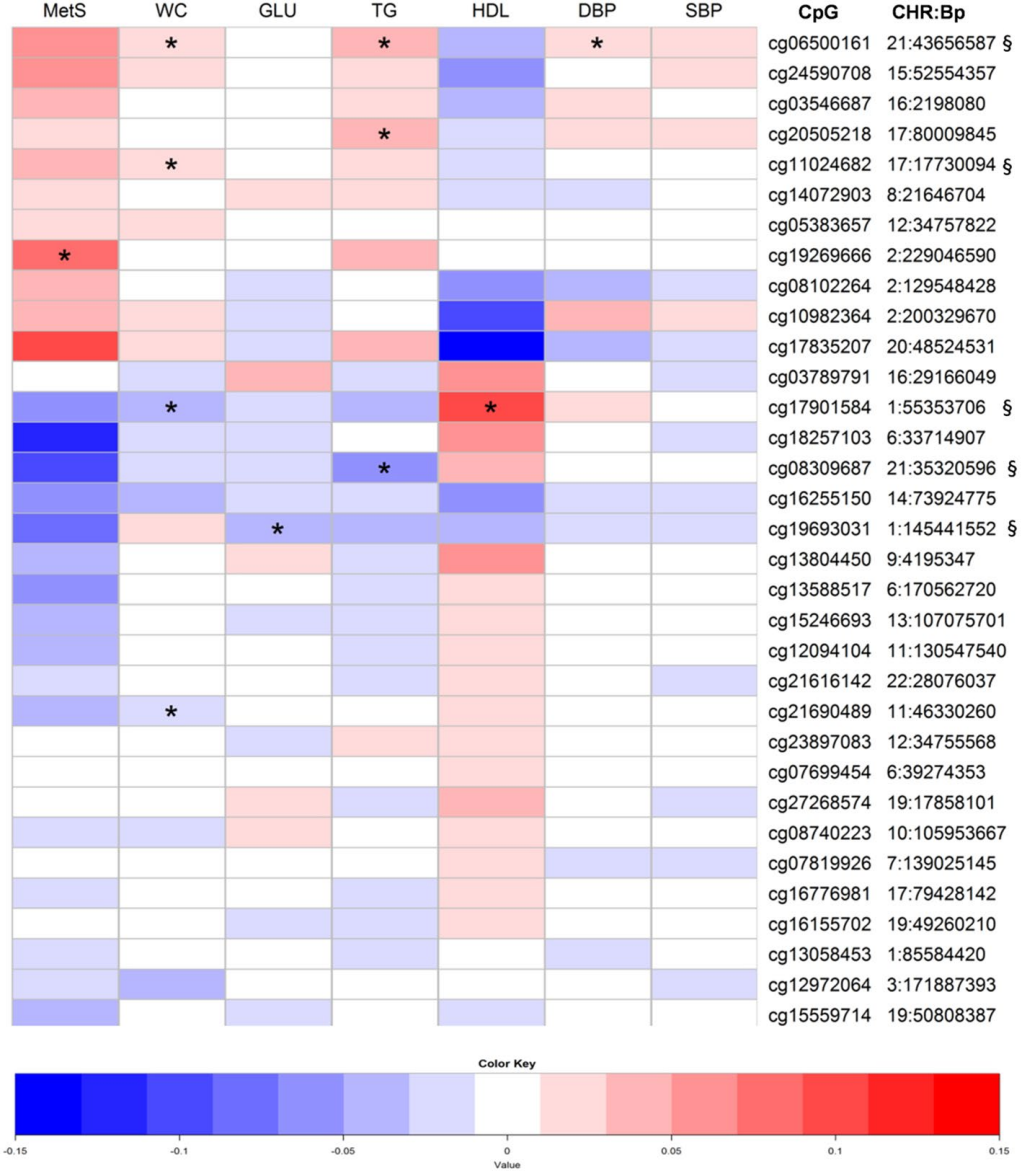
Association of CpG methylation sites across metabolic syndrome (MetS) and related phenotypes in the NFBC1966 cohort. The heat map includes the 33 most significant probes from the discovery epigenome-wide association studies (EWAS). Colours in the map refer to the effect size (beta) values for the probe in question. Beta values exceeding 0.15 are recoded as 0.15 and values below –0.15 are recoded as –0.15 in the figure. Stars indicate significant *P* values from the individual tests as follows: *P < 0.01, **P < 0.0001, ***P < 1 × 10^−6^. Beta values from the waist circumference, diastolic blood pressure, and systolic blood pressure analyses are multiplied by 10 in order to provide more comparable and clinically relevant values for beta in the heat map. The detailed numeric data for the heat map is presented in the Supplementary Table 2. § indicates the successful replication of the association between the methylation probe and the individual phenotype. *MetS* metabolic syndrome as case/control, *WC* waist circumference, *GLU* fasting glucose, *HDL* high-density lipoprotein, *TG* triglycerides, *DBP* diastolic blood pressure, *SBP* systolic blood pressure, *CpG* cytosine–guanine dinucleotide, *CHR* chromosome, *Bp* base pair.

Figure 1 shows some general behaviour patterns of MetS component phenotypes (Fig. 1). Notably, the association for the blood pressure components (systolic- and diastolic blood pressure) of MetS behaved rather independently compared to other components. Methylation probe cg19693031 in the *TXNIP* region emerged as the only replicated probe binding blood pressure to other MetS components. Interestingly, methylation site cg06500161 in the gene *ABCG1* appeared to also associate with diastolic blood pressure in addition to the association with triglycerides and WC in the replication cohort (Fig. 2, Supplementary Table 2), despite this association remaining unidentified in the discovery analysis (Fig. 1, Suppl. Table 1).

### Examination of the underlying genetic effects

To test the possible underlying genetic effects (DNA variation expressed as an SNP effect) behind the CpG–phenotype associations, we used as a covariate in the conditional analyses the strongest associating SNP ± 5 Mb from the genomic position of each replicated methylation probe for analysis of cg19693031 and glucose, cg17901584 and HDL, cg06500161 and triglycerides, and cg11024682, cg06500161 and cg08309687 and WC (Tables 3 and 4). Using this approach, we found suggestive evidence for a genetic effect in our analysis for cg19693031 in gene *TXNIP* and glucose as well as for cg11024682 in the gene *SREBF1* and WC. After adjusting for the genetic signal rs186387341, we found a stronger association (β_eff_ = –0.136, *P* = 1.69 × 10^−8^) between glucose and the cg19693031 methylation site. While adjusting for the genetic signal rs183499598, a stronger association (β_eff_ = 0.003, *P* = 5.94 × 10^−8^) between WC and the cg11024682 methylation site was observed (Table 4).

**Table 4:** Conditional analysis for the replicated CpG M value—phenotype associations.

Phenotype	CpG	Gene	SNP used for conditioning	Discovery analysis among DILGOM			Conditional analysis among DILGOM		
				Effect	SE	*P*	Effect	SE	*P*
Fasting glucose	cg19693031	*TXNIP*	rs186387341	− 0.123	0.023	8.47E−08	− 0.136	0.024	1.69E−08
HDL	cg17901584	*DHCR24/HP08874* mRNA, partial sequence	rs549430503	0.156	0.034	4.63E−06	0.164	0.035	3.25E−06
Triglycerides	cg06500161	*ABCG1*	rs188029560	0.069	0.015	3.49E−06	0.073	0.015	2.39E−06
WC	cg11024682	*SREBF1*	rs183499598	0.003	0.001	1.15E−07	0.003	0.001	5.94E−08
	cg06500161	*ABCG1*	rs192988322	0.004	0.001	1.75E−06	0.004	0.001	4.79E−06
	cg08309687	long intergenic non-protein coding RNA 649	rs183531367	− 0.005	0.001	1.85E−06	−0.005	0.001	3.69E−06

*DILGOM* dietary, lifestyle, and genetic determinants of obesity and metabolic syndrome, *WC* waist circumference, *CpG* cytosine–guanine dinucleotide, *SNP* single-nucleotide polymorphism, *SE* standard error.

### Associations of replicated DNA methylation loci and gene expression

We examined the relation of the replicated methylation loci annotated to a gene with gene expression of the gene in question. Gene expression data was available for transcripts of genes *TXNIP, SREB* and *ABCG*1 (Table 5). The methylation site cg11024682 annotated to *SREB* gene body associated inversely with *SREB* gene transcript (β_eff_ = −0.119, *P* = 0.045) indicating that the increased methylation was associated with decreased gene expression of the corresponding gene (Table 5). We could also see inverse borderline significant association between methylation site cg11024682 and *ABCG*1 gene transcript (β_eff_ = −0.185, *P* = 0.051) marking a possible *trans* effect between the methylation locus and the gene annotated to a different chromosome (Supplementary Table 4). The methylation site cg06500161 annotated to *ABCG*1 gene body showed a strong inverse association with *ABCG*1 gene transcript (β_eff_ = −0.221, *P* = 0.0043). The methylation site cg19693031 annotated to 3′UTR of gene *TXNIP* showed no association with *TXNIP* gene transcript (β_eff_ = −0.004, *P* = 0.95). Interestingly, we were able to see a suggestive direct *trans* effect between the methylation locus and the gene transcripts of *SREB* (β_eff_ = 0.062, *P* = 0.038) as well as two separate transcripts of *ABCG*1 (β_eff_ = 0.111, *P* = 0.0011; β_eff_ = 0.123, *P* = 0.0094) (Supplementary Table 4).

**Table 5:** Gene expression—CpG M value associations of successfully replicated CpG methylation sites.

CpG	Position (Chr:bp) GRCh37	Gene (CpG)	Strand	UCSC RefGene Group	Relation to UCSC CpG Island	Enhancer	Regulatory Feature Group	DMR	Transcript	*n*	Effect	SE	*P*
cg19693031	1:145,441,552	*TXNIP*	F	3′UTR	-	-	-	-	ILMN 1697448	512	−0.004	0.07	0.95
cg17901584	1:55,353,706	*DHCR24*	F	TSS1500	S_Shore	-	Promoter Associated Cell type specific	-	NA	NA	NA	NA	NA
cg11024682	17:17,730,094	*SREBF1*	R	Body	S_Shelf	-	Unclassified Cell type specific	-	ILMN 1663035	512	−0.119	0.06	0.045
									ILMN 1695378	512	0.087	0.06	0.14
cg06500161	21:43,656,587	*ABCG1*	F	Body	S_Shore	TRUE	-	-	ILMN 2329927	512	−0.221	0.08	0.0043
									ILMN 1794782	512	−0.095	0.06	0.088
									ILMN 1695968	512	0.038	0.04	0.32
									ILMN 1743638	512	0.017	0.03	0.61
									ILMN 2262362	512	0.014	0.03	0.66
cg08309687	21:35,320,596	-	F	-	-	TRUE	Promoter Associated	DMR	NA	NA	NA	NA	NA

*CpG* cytosine–guanine dinucleotide, *Chr* chromosome, *bp* base pair, *GRCh37* the Genome Reference Consortium human genome (build 37), *UCSC* University of California Santa Cruz, *DMR* differentially methylated region, *SE* standard error. Annotation information for CpG methylation sites is received from the annotation file of Illumina Infinium Human Methylation 450 BeadChip.

## Discussion

We performed EWAS of MetS and its components to shed light on the biological background of this complex phenotype. In an interpretation of the results the evident overlapping interrelation of separate component traits of MetS should be taken into account. In should also be noted that medication is in general a known epigenome altering factor. Nevertheless we believe that our analyses capture rather association signals induced by studied phenotypes than by individual drug ingredients used by study individuals (Supplementary material, Supplementary Table 3). Our data suggest that the interplay between lipid and glucose metabolism may represent a key element in the pathology of MetS, implying that epigenetic markers linking blood pressure to the other components of MetS may be scarce.

### The role of replicated methylation loci in metabolism

We observed numerous associations between CpG methylation sites and separate component phenotypes of MetS. The essential role of dyslipidemia in MetS was previously suggested^6^. Our data support the view that the biological background of MetS strongly points towards alterations in lipid metabolism. Methylation site cg06500161 in gene *ABCG1* associated with triglycerides and WC in our primary EWAS and suggested an association with HDL, glucose, and MetS as a condition. We also saw a clear inverse correlation between the methylation locus and the gene expression of *ABCG1* indicating the possible changes in gene function related to the methylation of cg06500161. The association between cg06500161 and triglyceride and HDL metabolism was previously reported, and the locus appears to act as an epigenetic link between myocardial infarction and the blood lipid levels^25^. In addition, the methylation site was previously found to act as an integral part of insulin and glucose metabolism, as well as associating with homeostatic model assessment for insulin resistance (HOMA-IR), a commonly used surrogate to define the state of insulin resistance^26,27^. Cg06500161 is also directly associated with type 2 diabetes, and appears to serve as an epigenetic marker when evaluating an individual’s risk for it^28^. Recent study directly linked the site to MetS^10^. Our data support the idea that the *ABCG1* gene plays an important role in both lipid and glucose metabolism, suggesting that it could potentially link the two as an underlying factor in the pathology of MetS. In a recent study, support for the hypothesized role of body mass index as a factor partly explaining the association between type 2 diabetes and DNA methylation was given^29^. Our data suggests that these findings can be seen in a broader context of MetS -related dyslipidaemia as a preceding state of type 2 diabetes.

Cg11024682 in the *SREBF1* gene associated with WC in our primary analysis suggesting an association with triglycerides and MetS. The association between cg11024682 and triglycerides has been previously identified, whereby the results were validated through a tissue-specific analysis using adipose and skin tissue^25^. In addition, an interaction between the gene products of *ABCG1* and *SREBF1* has also been reported^25^. Furthermore, evidence exists for the role of *SREBF1* in glucose metabolism and type 2 diabetes^26,28,30^. While we found no association between glucose metabolism and *SREBF1* in our analyses, our findings link the function of *SREBF1* to MetS, thus supporting the concept of an altered lipid metabolism related to the syndrome. The possible effect of cg11024682 methylation locus on expression of *SREBF1* was supported by our findings. Our suggestive finding about the possible *trans* effect between the methylation locus at *SREBF1* and the expression of *ABCG1* further support the idea about the interaction between the two genes.

The third replicated methylation site associated with WC in our data, cg08309687 located in the intergenic area downstream from the *ABCG1* gene region, suggested a correlation with triglyceride levels. Interestingly, in a family study attempting to identify novel epigenetic determinants of type 2 diabetes^28^, this methylation site represented one of the most significant CpG sites directly associated with type 2 diabetes, along with fasting glucose and insulin resistance. To our knowledge, our study is the first to also link this methylation site to lipid metabolism.

Additionally, we report the novel association between cg17901584 in chromosome 1 and HDL as well as the association between this methylation site and triglycerides and WC. This is, to our knowledge, the first time that cg17901584, located approximately 1 kb downstream from 24-dehydrocholesterol reductase (*DHCR24*) has been linked to lipid metabolism at the methylation level.

The methylation site cg19693031 in the gene *TXNIP* was previously found to associate with lipid metabolism^31^, and its role in glucose metabolism and type 2 diabetes has been confirmed in many recent studies^28,30,32,33^. Our data also indicates that cg19693031 is indeed an important methylation site, possibly linking lipid and glucose metabolism to each other. In our association analyses between cg19693031 methylation locus and gene expression we could see that rather than affecting the expression of its “own” gene *TXNIP* the locus shows association with the expression of lipid associated genes *SREBF1* and *ABCG1*. Notably, in our analyses, cg19693031 was the only successfully replicated methylation site binding all of the different components together with MetS. In our search for methylation loci linking the individual MetS components together as a pathological cardiometabolic condition, cg19693031 in the *TXNIP* gene appears to represent a strong candidate.

To date, only a few studies have investigated the epigenome-wide association for MetS as a condition^8,10^. In a recent study, methylation sites cg00574958 and cg17058475, both located on chromosome 11, associated with MetS^8^. In our study, we reproduced this result at a significance level of *P* < 0.05 (β = −0.08, *P* = 4.56 × 10^−3^ for cg00574958; β = –0.09, *P* = 5.21 × 10^−3^ for cg17058475 in the discovery analysis). In another study^10^, methylation site cg06638433 on chromosome 17 associated with MetS alongside the cg06500161. We were unable to reproduce the result for cg06638433 at a significance level of *P* < 0.05 (β = 0.02, *P* = 2.85 × 10^−1^).

### Blood pressure as a component of MetS

Our data identified the rather independent behaviour of blood pressure components on MetS compared to the metabolic components of the syndrome, as well as the non-aligned behaviour between diastolic and systolic blood pressure (Fig. 1). Only one of the four probes showing suggestive association with blood pressure on top of the other components of MetS, cg19693031, was replicated. Thus, any successfully replicated connective epigenetic marker can serve as a solid target in future studies of the MetS biology. We should also note that the mean age of the replication cohort of our study is rather low, possibly explaining the small replication rate among the blood pressure–related probes in our analyses (Table 1). At the genetic level, the association between the *TXNIP* allele variation, type 2 diabetes, and hypertension was previously demonstrated, and the role of *TXNIP* as a key modulator linking the diabetogenic and vascular pathways behind metabolic conditions has been discussed^34,35^. To our knowledge, however, our study is the first to suggest that the methylation component should also be considered in such discussions.

In our study, the non-replicated methylation probe cg07699454 appeared to associate with MetS and its lipid components, as well as with diastolic blood pressure. This probe is located in the gene *KCNK17,* which has previously been associated with a risk of stroke^36^. It also resides in close proximity to the gene *KCNK16*, a known susceptibility locus for type 2 diabetes^37^. These findings render cg07699454 an interesting locus for future studies on the role of vascular components of type 2 diabetes and MetS. The apparent association between cg06500161 and diastolic blood pressure in NFBC1966 represents an interesting finding. The well-characterised *ABCG1* gene has only very recently been associated with vascular phenotypes^38^. Our data also support the previous idea that cg06500161 could represent a target locus in further studies regarding the molecular background of MetS.

### Strengths and limitations

Our study has several strengths. Finnish population cohorts are, in general, homogeneous, both genetically and culturally and well characterised, making biological patterns detectable even amongst relatively small sample sizes. In addition, a detailed cohort characterisation also helped to construct analytical models in a harmonious way, which is crucial when attempting to control the challenging nature of methylation data. We observe a slightly increased pattern of general hypomethylation in association analyses performed in the discovery cohort with older individuals, compared to the younger replication cohort. The finding, even though should be examined with a caution, fits well with the general observation about promoting effect of aging leading to a global genome-wide hypomethylation in various types of tissues. We should, however, address some limitations. As discussed in a wide range of EWASes, whole-blood peripheral white blood cells are not ideal sources of methylation data, since methylation as a DNA regulation mechanism is highly tissue-specific. Furthermore, due to methylation’s great sensitivity to variation in age, our replication cohort may not be the best possible cohort for the purpose and may in part explain the relatively modest success in replication of our findings. However, because of the notable age- difference between discovery and replication cohorts, the statistical power of detecting true positive findings diminishes. Thus we feel that the risk of detecting and reporting false positive findings in our study remains small. We also recognise the role of elevated waist circumference as a prerequisite of MetS condition in 2005 IDF definition for MetS, and it cannot be ruled out that associations that we see between different methylation sites and MetS might for one manifest the association between methylation site and waist circumference.

## Conclusions

Taken together, our study links the previously type 2 diabetes–associated methylation locus cg08309687 to lipid metabolism. We also found a novel association between the methylation locus cg17901584 and HDL. Overall, our study supports the idea derived from genetic studies whereby aberrations in lipid metabolism, combined with the interplay with glucose metabolism, play central roles as functional elements of MetS. In our study we consider EWAS as a tool of a hypothesis free approach to examine if epigenetic variation explains any variation in the phenotype to be studied. The epigenetics of MetS appear polymorphic as expected. Our data suggest that blood pressure might act rather independently compared to the metabolic components of MetS, at least at the epigenetic level. In our attempt to identify a comprehensive methylation locus behind the MetS condition, cg19693031 in gene *TXNIP* emerges as a strong candidate.

## Supplementary information

Supplementary Information.

Supplementary Information 2.

Supplementary Information 3.

## Data Availability

The data that support the findings of this study are available from THL Biobank and University of Oulu but restrictions apply to the availability of these data, which were used under license for the current study, and so are not publicly available. Data are however available from the authors upon reasonable request and with permission of THL Biobank and University of Oulu.
