# Supplementary material for: An epigenome-wide association study of metabolic syndrome and its components
Source: Sci Rep. 2020 Nov 25;10:20567. doi: 10.1038/s41598-020-77506-z (PMC7688654; doi:10.1038/s41598-020-77506-z)

**An Epigenome-Wide Association Study of Metabolic Syndrome and Its Components**

Marja-Liisa Nuotio, Natalia Pervjakova, Anni Joensuu, Ville Karhunen, Tero Hiekkalinna, Lili Milani, Johannes Kettunen, Marjo-Riitta Järvelin, Pekka Jousilahti, Andres Metspalu, Veikko Salomaa, Kati Kristiansson, and Markus Perola

**Supplementary Material**

**Contents:**

Methodologies in sample phenotyping

Sample size and technical covariates in EWAS analyses

Supplementary Table 1—Results of the discovery EWAS of CpG methylation probes that were selected for replication. Please see separate Excel file.

Supplementary Table 2—Results of the replication EWAS of CpG methylation probes that were selected for replication. Please see separate Excel file.

Supplementary Table 3—Effect of different drug ingredients on discovery analysis results for top findings.

Supplementary Table 4— Gene expression - CpG M value associations between non-equivalent gene transcripts and CpG methylation sites.

Supplementary Figure 1 —Q–Q probability plots generated from the results of the discovery EWAS.

Supplementary Figure 2—Q–Q probability plots generated from the results of the association analyses between SNPs and replicated CpG M value - phenotype associations to allow the selection of SNPs used for the conditioning in conditional analyses.

**Methodologies in sample phenotyping**

**the National FINRISK Study, DILGOM**

In DILGOM cohort, blood pressure measurements were performed by trained nurses with the mercury manometer in sitting position from the right arm of the individual after at least 5 minutes of rest. The measurement was repeated three times, with the intermission period of 1 minute. The final variable of systolic and diastolic blood pressure was defined to be the average of the 2nd and 3rd measurement.

Waist circumference (WC) was measured in standing position with the tape positioned at a level midway between the lower rib margin and iliac crest.

Blood sampling was performed in the fasting state, by choice after at least 10h duration of fast and stored at -80°C in a freezer. Measurements from blood sample were executed 2-3 months after sampling. Triglycerides were measured with an enzymatic colorimetric determination (GPO-PAP method). HDL-cholesterol measurements were performed with a direct homogenous enzymatic colorimetric determination (CE-CO-POD method). Fasting blood glucose was measured with a glucose hexokinase method.

For information about medical history and medication, participants filled a specific questionnaire.

**The Northern Finland Birth Cohort 1966 (NFBC1966)**

In NFBC1966, anthropometric measurements and blood pressure measurements were performed by trained nurses using a standard mercury sphygmomanometer after a 15-min rest period.

Blood samples were drawn after overnight fasting. Samples were stored at –70°C until analysed.

Fasting serum glucose, HDL, and triglycerides were determined using a Hitachi 911 automatic analyser and commercial reagents (Boehringer Mannheim, Mannheim, Germany).

**Sample size and technical covariates in epigenome-wide association analyses**

In DILGOM, the overall sample size 517 refers to the individuals for whom methylation data were available. Sample sizes vary between analysed traits according to the number of individuals for whom the data for specific trait were available. Before the performance of epigenome-wide association analyses, the calculation of summary statistics was performed for each phenotype trait to be analysed, after which the individual phenotype measurements exceeding the value of standard deviation multiplied by four were excluded from analyses as outliers. Also gravid individuals were excluded from analyses. After exclusions the number of individuals left for discovery associations analyses was 496 for MetS, 498 for triglycerides, 498 for HDL-cholesterol, 498 for fasting blood glucose, 497 for waist circumference and 497 for both, systolic and diastolic blood pressure. Among the MetS cases of DILGOM five individuals had experienced angina pectoris symptoms during last 12 months at a time of data collection, seven individuals had diagnosis of myocardial infarction and one individual had balloon angioplasty performed without a known cause (at our data collection). Among MetS controls, the equivalent numbers were four individuals for angina pectoris and two for diagnosis of MI. Among MetS cases, 78 individuals used medication for hypertension, 47 individuals used medication for aberrations in blood lipids and 14 individuals were medicated for increased levels of blood glucose. Among MetS controls, the equivalent numbers were 28, 25 and two, respectively.

In NFBC1966, out from the total sample with DNA methylation data, one outlier and seven gender mismatches were removed before association analyses.

Epigenome-wide association analyses for discovery cohort were adjusted for age, sex, smoking status (defined either as current or never/ex-smokers), alcohol consumption (grams/week), cell subtype proportion (CD4+ T cells, CD8+ T cells, NK cells, granulocytes, monocytes), chip-ID and position on methylation chip, the first 30 principal components from the principal component analysis performed for HM450K control probes and the first five genetic principal components of the data to control for potential population substructure.

Epigenome-wide association analyses for replication cohort were adjusted for age, sex, smoking status (defined either as current or never/ex-smokers), alcohol consumption (grams/week), cell subtype proportion (CD4+ T cells, CD8+ T cells, NK cells, granulocytes, monocytes), the first 30 principal components from the principal component analysis performed for HM450K control probes and the first five genetic principal components of the data to control for potential population substructure.

Supplementary Table 3—Effect of different drug ingredients on discovery analysis results of top findings.

|  |  |  |  |  | **DILGOM** | | | |
| --- | --- | --- | --- | --- | --- | --- | --- | --- |
| **Phenotype** | **Analysis model** | **CpG** | **Position (Chr:bp)**  **GRCh37** | **Gene** | ***n*** | **Effect** | **SE** | ***P*** |
| Fasting glucose | original | cg19693031 | 1:145441552 | TXNIP | 498 | - 0.123 | 0.023 | 8.47E-08 |
|  | original + full medication * | cg19693031 | 1:145441552 | TXNIP | 498 | - 0.080 | 0.025 | 1.76E-03 |
|  | original + medication for elevated blood pressure | cg19693031 | 1:145441552 | TXNIP | 498 | - 0.113 | 0.023 | 1.14E-06 |
|  | original + medication for elevated serum lipids | cg19693031 | 1:145441552 | TXNIP | 498 | - 0.125 | 0.023 | 8.54E-08 |
|  | original + medication for elevated blood glucose | cg19693031 | 1:145441552 | TXNIP | 498 | - 0.089 | 0.025 | 3.82E-04 |
| WC | original | cg11024682 | 17:17730094 | SREBF1 | 497 | 0.003 | 0.001 | 1.15E-07 |
|  | original + full medication * | cg11024682 | 17:17730094 | SREBF1 | 497 | 0.003 | 0.001 | 5.27E-08 |
|  | original + medication for elevated blood pressure | cg11024682 | 17:17730094 | SREBF1 | 497 | 0.003 | 0.001 | 3.47E-08 |
|  | original + medication for elevated serum lipids | cg11024682 | 17:17730094 | SREBF1 | 497 | 0.003 | 0.001 | 1.65E-07 |
|  | original + medication for elevated blood glucose | cg11024682 | 17:17730094 | SREBF1 | 497 | 0.003 | 0.001 | 1.55E-07 |
|  | original | cg06500161 | 21:43656587 | ABCG1 | 497 | 0.004 | 0.001 | 1.75E-06 |
|  | original + full medication * | cg06500161 | 21:43656587 | ABCG1 | 497 | 0.003 | 0.001 | 2.28E-05 |
|  | original + medication for elevated blood pressure | cg06500161 | 21:43656587 | ABCG1 | 497 | 0.003 | 0.001 | 6.82E-06 |
|  | original + medication for elevated serum lipids | cg06500161 | 21:43656587 | ABCG1 | 497 | 0.004 | 0.001 | 2.58E-06 |
|  | original + medication for elevated blood glucose | cg06500161 | 21:43656587 | ABCG1 | 497 | 0.003 | 0.001 | 6.88E-06 |

DILGOM, Dietary, Lifestyle, and Genetic Determinants of Obesity and Metabolic Syndrome; NFBC1966, Northern Finland Birth Cohort 1966; HDL, high-density lipoprotein; WC, waist circumference; CpG, cytosine–guanine dinucleotide; Chr, chromosome; bp, base pair; GRCh37, the Genome Reference Consortium human genome (build 37); SE, standard error. * Full medication indicates the adjustment of original discovery association analyses for fasting glucose and waist circumference with medication for elevated blood pressure, medication for elevated serum lipids and for elevated blood glucose. Aforementioned analyses are also adjusted separately with each of the three different medication groups. Results in Supplementary Table 3 are presented for top findings of the EWA-study.

Supplementary Table 4— Gene expression - CpG M value associations between non-equivalent gene transcripts and CpG methylation sites.

| **Expressed Gene** | **Position (Chr:bp)**  **GRCh37** | **Transcript** | **CpG** | **Position (Chr:bp)**  **GRCh37** | **Gene (CpG)** | ***n*** | **Effect** | **SE** | ***P*** |
| --- | --- | --- | --- | --- | --- | --- | --- | --- | --- |
| *ABCG1* | 21:43656587 | ILMN 2329927 | cg11024682 | 17:17730094 | *SREBF1* | 512 | -0.185 | 0.09 | 0.051 |
| *SREBF1* | 17:17730094 | ILMN 1663035 | cg19693031 | 1:145441552 | *TXNIP* | 512 | 0.062 | 0.03 | 0.038 |
| *ABCG1* | 21:43656587 | ILMN 1794782 | cg19693031 | 1:145441552 | *TXNIP* | 512 | 0.111 | 0.03 | 0.0011 |
| *ABCG1* | 21:43656587 | ILMN 2329927 | cg19693031 | 1:145441552 | *TXNIP* | 512 | 0.123 | 0.05 | 0.0094 |

Chr, chromosome; bp, base pair; GRCh37, the Genome Reference Consortium human genome (build 37); CpG, cytosine–guanine dinucleotide; SE, standard error.

Supplementary Figure 1 —Q–Q probability plots generated from the results of the discovery EWAS.


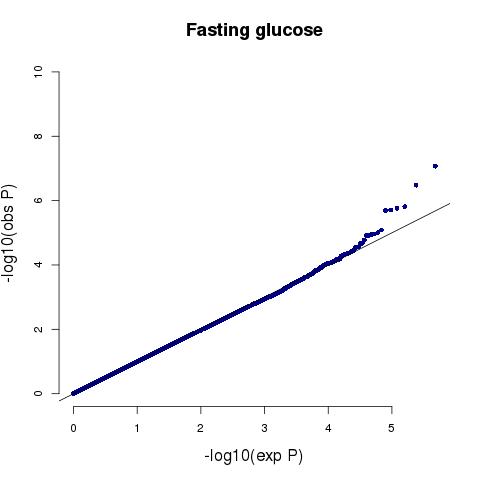

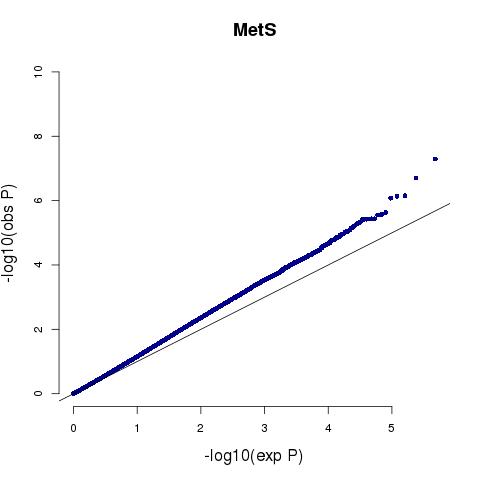


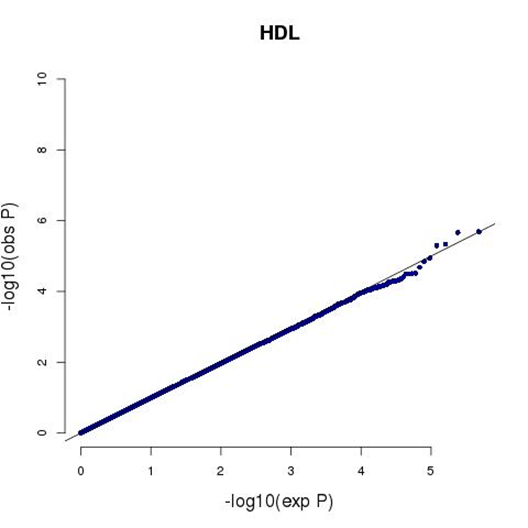

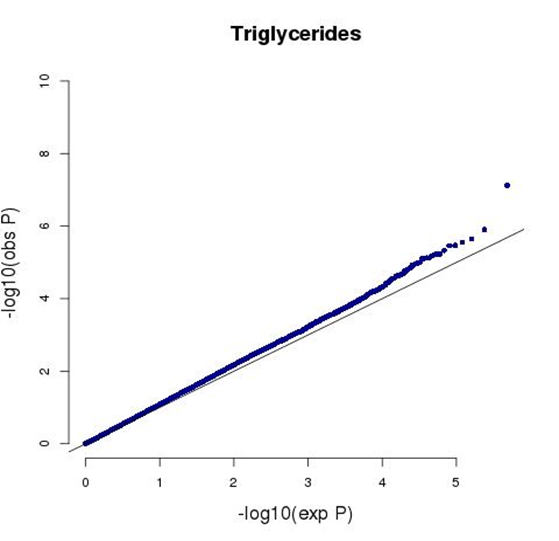


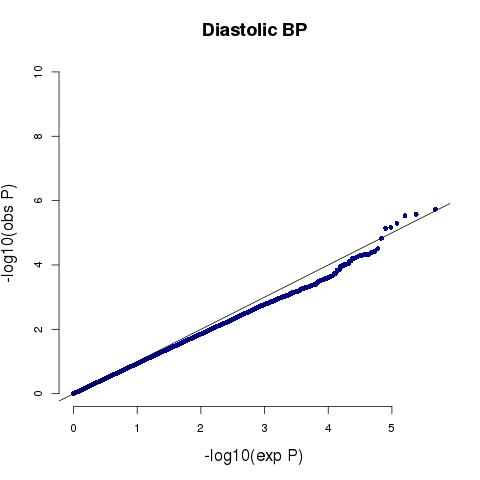

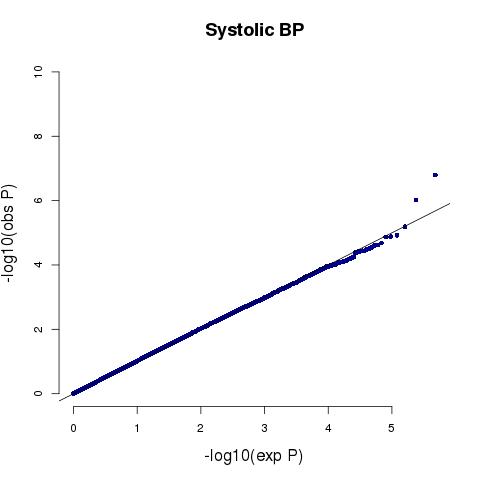


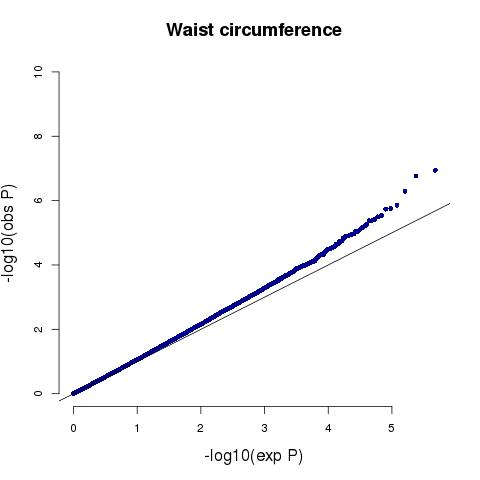


Supplementary Figure 2—Q–Q probability plots generated from the results of the association analyses between SNPs and replicated CpG M value - phenotype associations to allow the selection of SNPs used for the conditioning in conditional analyses.


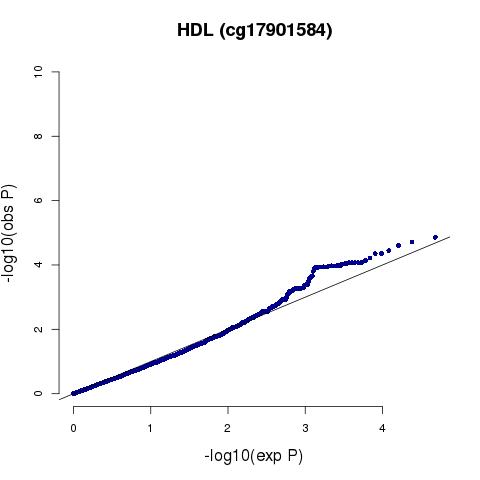

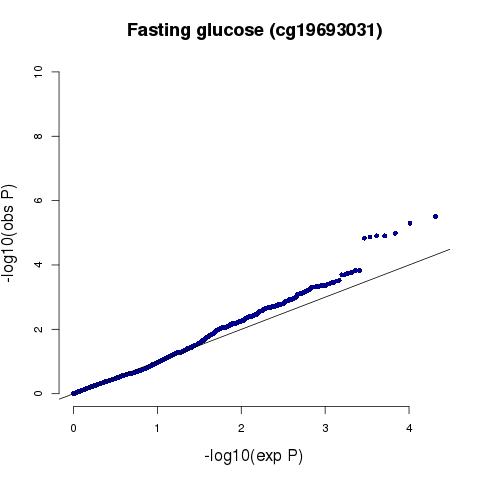


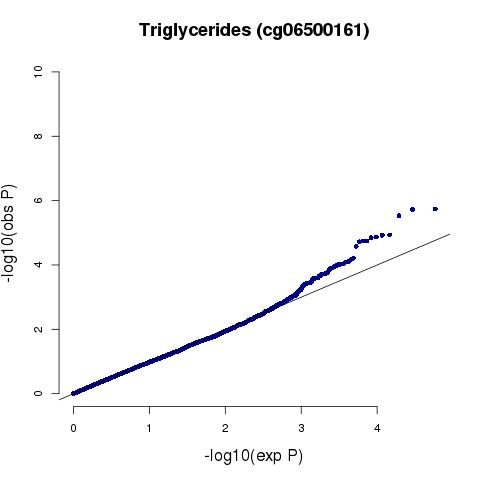


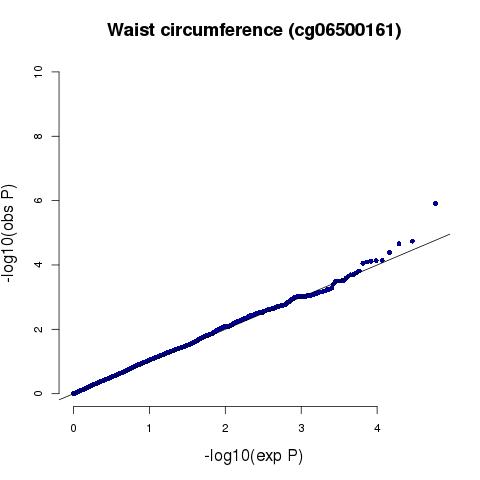

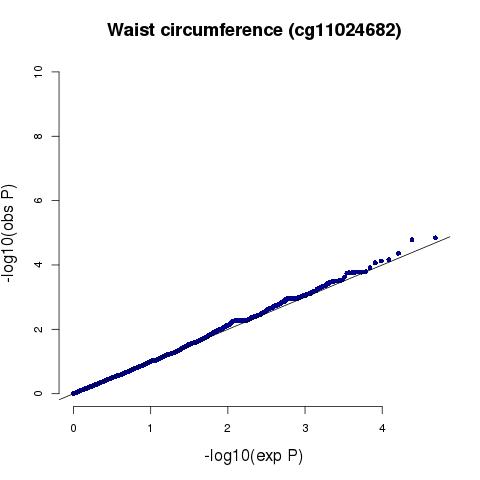


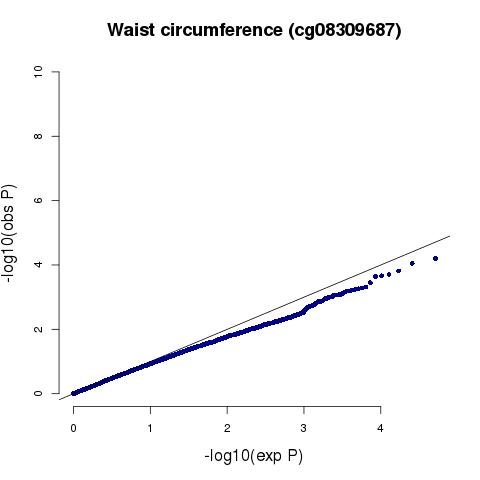

Supplement: Supplementary file 1 — Supplementary Information. [file 41598_2020_77506_MOESM1_ESM.docx]
